# A Novel Tetrahydrocannabinol Electrochemical Nano Immunosensor Based on Horseradish Peroxidase and Double-Layer Gold Nanoparticles

**DOI:** 10.3390/molecules21101377

**Published:** 2016-10-17

**Authors:** Dingqiang Lu, Fuping Lu, Guangchang Pang

**Affiliations:** 1 College of Biotechnology, Tianjin University of Science &Technology, Tianjin 300457, China; m18222368363@163.com (D.L.); lfp@tust.edu.cn (F.L.); 2 Key Laboratory of Industrial Fermentation Microbiology, Ministry of Education, Tianjin 300457, China; 3 College of Biotechnology & Food Science, Tianjin University of Commerce, Tianjin 300314, China; 4 Tianjin Key Laboratory of Food Biotechnology, Tianjin 300314, China

**Keywords:** tetrahydrocannabinol (THC), electrochemical, immunosensor, amperometric I-t curve method, gold nanoparticles (GNPs)

## Abstract

In the current study, a novel double-layer gold nanoparticles-electrochemical immunosensor electrode immobilized with tetrahydrocannabinol (THC) antibody derived from Balb/c mice was developed. To increase the fixed quantity of antibodies and electrochemical signals, an electrochemical biosensing signal amplification system was utilized with gold nanoparticles-thionine-chitosan absorbing horseradish peroxidase (HRP). In addition, a transmission electron microscope (TEM) was used to characterize the nanogold solution. To evaluate the quality of the immunosensor, the amperometric I-t curve method was applied to determine the THC in PBS. The results showed that the response current had a good linear correlation with the THC concentration range from 0.01~10^3^ ng/mL with a correlation coefficient of 0.9986. The lowest detection limit for THC was 3.3 pg/mL (S/N = 3). Moreover, it was validated with high sensitivity and reproducibility. Apparently, the immunosensor may be a very useful tool for monitoring the THC.

## 1. Introduction

There are over 70 kinds of cannabinoids separated from dry matters of hemp and fresh leaves of hemp, in which the amount of tetrahydrocannabinol (THC) is the highest. THC is well-known as a drug and is the secondary metabolite of the special molecular structure with alkyl and monoterpene perssad in hemp. It is the major component in marijuana which influences mental state, makes people have hallucinations, and become addicted [1,2]. Besides, THC is mainly used for treating some diseases which other medicines have poor effects on, such as multiple sclerosis (MS), motor nerve diseases, chronic refractory pain, and drug-induced emesis of nervous system diseases [3,4,5,6,7]. It may have a certain effect on glaucoma, asthma, and cardiovascular diseases [8,9]. Therefore, rapid detection of THC with more sensitive approaches is of great importance to human health, drug control, and detection.

To explore the rapid, accurate, simple, and highly sensitive detection method, a large number of studies on detection of THC have been conducted. The usual methods for detecting THC are test strip/board/box (gold immuno-chromatography assay, GICA) and enzyme-linked immunoassay (ELISA) which can achieve the goal of a qualitative test. GICA is simple and quick, but it can only be used as a primary screening method due to the limit of sensitivity (the sensitivity is about 50 ng/mL). Compared with GICA, ELISA has significant improvements in sensitivity (about 1 ng/mL) and specificity. However, immunoassays are subject to interference and may generate false positive screening results. In addition, physicochemical detection methods such as high performance liquid chromatography (HPLC) and gas chromatography-mass spectrometry method (GC/MS) are generally widely recognized as affirming methods for detecting THC because of good sensitivity and specificity. Time-consuming pre-column derivatization is unavoidable, and both HPLC and GC/MS shall be done in the laboratory and be carried out by professional technologists through facilities at high costs [10,11,12,13,14].

In recent years, electrochemical immunosensors have drawn a lot of attention for research. Why? On one hand, the electrochemical sensor technology has been very mature and widespread. This technology can turn the weak signal of a combination of antigen and antibody into an electrochemical signal. After amplification and treatment, the quick and quantitative detection for antigens or pathogenic bacteria with the antigen can be realized through electrochemical system [15]. On the other hand, the use of Balb/c mouse monoclonal antibody also helps. Theoretically, all the biomacromolecules like protein [16] and microorganisms [17] which can be used as antigens, and compounds like toxins [18], hormones [19], antibiotics [20], and pesticides [21] which can be used as haptens can both be used to produce specific recognition antibodies, thus realizing the specific, quick, and highly sensitive immunodetection. The research results are respectively reported. Yang et al. [22] used single-wall carbon nanotubes with the nanogold to test the serum interleukin-6 (IL-6), indicating a wide linearity range and ultrahigh sensitivity: 0.01~100 fg/mL. Wang et al. [23] used a silicon-dioxide electrochemical immunosensor modified with nanogold to detect carcinoembryonic antigens (CEAs). The result shows that it has a wide linear relationship (10^−5^~10^2^ ng/mL) and the lowest detection limit up to 3.3 fg/mL with rather better selectivity, acceptable reproducibility, and acceptable stability. There is a specific combination mode between nano-materials and Balb/c mouse monoclonal antibody. It is generally acknowledged that the negative charge on the surface of nanogold adsorbs the perssad of positive charge of antibodies because of electrostatic interaction, and forms stable Au-S chemical bonds and other effects [24,25]. Tang et al. [26] find that nanogold mainly absorbs the antibody’s Fc terminal, and the rate of adsorption is 92%. The Fab terminal of the binding site to antigen is exposed to the outside, and can have specific reactions with antigen, which indicates that Balb/c mouse monoclonal antibody can be used in the detection of all kinds of biomolecules as an adaptor molecule.

In this study, a novel electrochemical immunosensor is developed with THC monoclonal antibodies (derived from Balb/c mice) as the recognition element. The chitosan (Chit)/gold nanoparticle (GNP) amplification system, consisting of horseradish peroxidase (HRP) and thionine (Thi), is constructed in this immunosensor. Through the fabricated immunosensor, THC could be detected successfully with high sensitivity and selectivity. As far as we know, this is a report on developing an electrochemical immunosensor to detect THC for the first time.

## 2. Results and Discussion

### 2.1. The Characterization of GNPs

**Figure 1:**
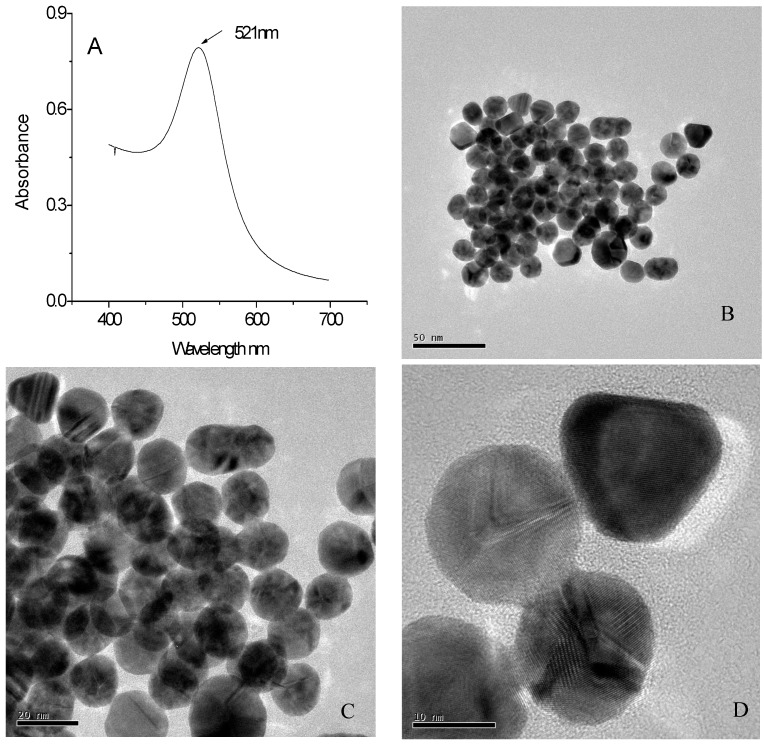
Spectral absorption curve of GNPs (**A**); TEM images of GNPs 1 × 71,000 (**B**); 1 × 145,000 (**C**); and 1 × 400,000 (**D**) respectively.

Nanomaterials like GNPs possess good conductivity, larger surface area, and good biocompatibility, which is helpful in the immobilization of an increased number of biomolecules to help reach the goal of amplifying electrochemical signals. The GNPs synthesized in this research have a bright red wine color. The spectral scanning of synthetic gold nanoparticles within the 400~700 nm wavelength range is shown in Figure 1A. Based on a strong absorption peak noted at 521 nm, and it can be roughly determined that the average particle size of the GNPs synthetised in this research is 15~20 nm. The transmission electron microscope (TEM) result of GNPs is as shown in Figure 1B–D. From the figures, it can be known that the GNPs synthesized in this research have a regular shape and uniform size with an average particle size of approximately 15 nm, but no aggregation. The UV-Vis characterization of GNPs is consistent with its TEM result, both of which have indicated that the particle sizes of the GNPs are 15~20 nm, and can be well used in the subsequent research.

### 2.2. The Characterization of Electrode Pretreatment

**Figure 2:**
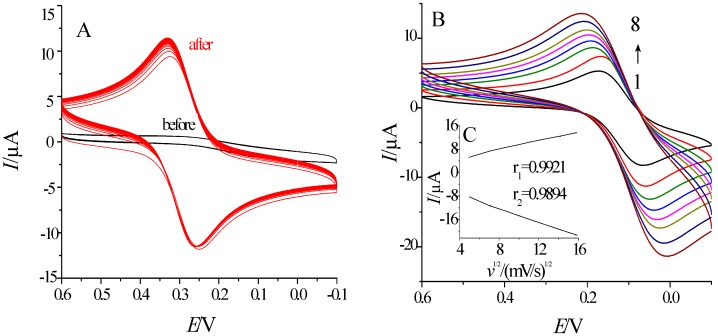
Characterization of the effect of the GCE pretreatment: (**A**) Cyclic Voltammetry; (**B**) Cyclic voltammograms of bare GCE at scan rates of 0.025, 0.050, 0.075, 0.100, 0.125, 0.150, 0.200, 0.250 V/s (1→8); the inset shows the dependence of the redox peak currents on the square root of scan rates.

In electrochemical immunoassay, cyclic voltammetry (CV) is the most commonly used method. The cyclic voltammogram of the glassy carbon electrode (GCE) before and after the pretreatment are as shown in Figure 2A. The activation of the glassy carbon electrode by H_2_SO_4_ could produce negatively charged oxygen-containing groups (such as hydroxyl and carboxyl, etc.) on its surface [27]. In addition, porous structure could form on the electrode surface and thereby increase its effective surface area through the pretreatment [28]. After the pretreatment, its redox peak current increases significantly. The peak-potential-difference is lower than 80mV and the peak current ratio is approximately 1, which indicates that the electrode conforms to the requirements; Figure 2B is the cyclic voltammogram of the electrode at different scan rates (1→8 are 25 mV/s, 50 mV/s, 75 mV/s, 100 mV/s, 125 mV/s, 150 mV/s, 200 mV/s and 250 mV/s) (scan range of 0.6~−0.1 V). According to the embedded diagram C in Figure 2B, the redox peak current had a good linear correlation with the square root of the scan rate, demonstrating that the redox peak current of the electrode was controlled only by diffusion. Therefore, it can be seen that the pretreatment effect of the glassy carbon electrode was good, and the electrode activated in H_2_SO_4_ would have improved performance and be useable in follow-up studies.

### 2.3. The Assembly and Characterization of the Electrode

**Figure 3:**
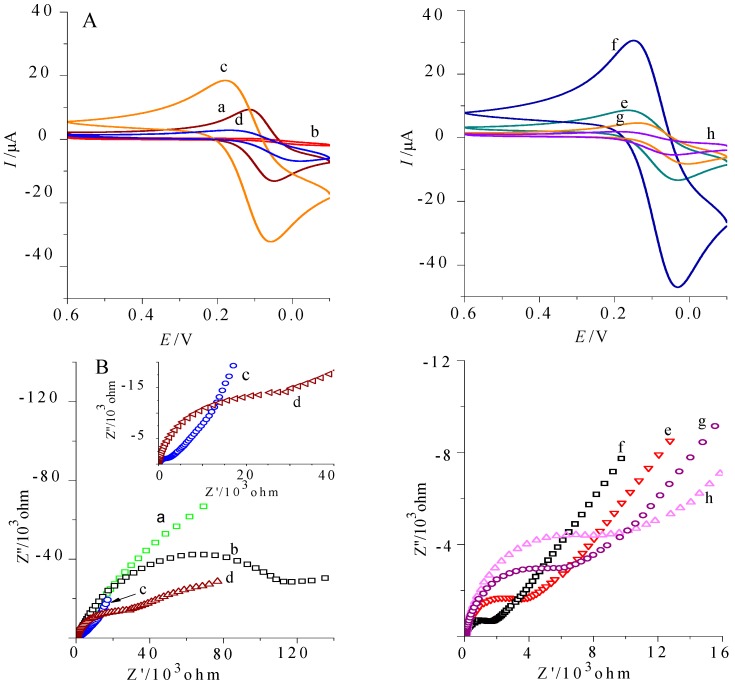
Characterized electrode in modifying process by Cyclic Voltammetry (**A**) and AC impedance (**B**) a–h: GCE, Chit-GCE, GNPs-Chit-GCE, Ab-GNPs-Chit-GCE, Thi/Chit-Ab-GNPs-Chit-GCE, HRP/GNPs-Thi/Chit-Ab-GNPs-Chit-GCE, Ab-HRP/GNPs-Thi/Chit-Ab-GNPs-Chit-GCE, BSA-Ab-HRP/GNPs-Thi/Chit-Ab-GNPs-Chit-GCE. Abbreviations: GCE, glassy carbon electrode; Chit, chitosan; GNP, Gold nanoparticles; HRP, horseradish peroxidase; Thi, thionine; Ab, antibody; BSA, bovine serum albumin.

Figure 3 showed the characterizations of different assembly stages of THC immunosensor in 1 mM K_3_Fe(CN)_6_ solution (containing 0.20 mol/L KNO_3_). Figure 3A represented cyclic voltammogram with the scan range of 0.6~−0.1 V and the scan rates of 50 mV/s; Figure 3B referred to AC impedance characterization with the scan range of 0.01~10^5^ Hz. The curve a, b, c, d, e, f, g, and h in the figure respectively represent eight different modification phases of bare GCE, Chit-GCE, GNPs-Chit-GCE, Ab-GNPs-Chit-GCE, Thi/Chit-Ab-GNPs-Chit-GCE, HRP/GNPs-Thi/Chit-Ab-GNPs-Chit-GCE, Ab-HRP/GNPs-Thi/Chit-Ab-GNPs-Chit-GCE, and BSA-Ab-HRP/GNPs-Thi/Chit-Ab-GNPs-Chit-GCE. From comparisons between the curves a and b, it can be seen that when chitosan was assembled on the bare GCE, the peak current of the cyclic Voltammetry significantly decreased and the impedance increased significantly. This was due to the electron transfer hindrance of the chitosan membrane, demonstrating the successful assembly of chitosan membrane. In the curve c, it can be seen that the redox peak current increased rapidly, and the impedance was drastically reduced. These changes were caused by the tunneling effect and the excellent electron transfer capability of gold nanoparticles to accelerate electron transfer. Compared with curve c, a significant decrease of redox peak current and the obvious increase of impedance shown in the curve d was ascribed to the low electrical conductivity of antibody molecules and the steric-hindrance effect on the electrode, indicating that the antibody was successfully assembled. Because the promotion of Thi for electron transfer was greater than the hindrance of chitosan on electron transfer, curve e showed a slight increase of redox peak current and a slight decrease in impedance compared with curve d. Curve f is the electrochemical characterization map after the assembly of the second layer of gold nanoparticles (absorbed with HRP) where the redox peak current increased significantly and the impedance was significantly reduced, indicating the promotion of gold nanoparticles for electron transfer was greater than steric hindrance of HRP on electron transfer. Compared with curve f, the redox peak current in curve g was significantly reduced and the impedance increased, which suggested that the second layer of antibody had been assembled on the electrode. In curve h, the redox peak current further reduced and the impedance further increased, which showed that BSA had blocked the non-specific sites on the electrode surface. Herein, double-layer anti-THC was used to increase the effective amount of antibody molecules. Crosslinked Chit containing Thi forming reticular structure were immobilized on GCE, which could adsorb and increase the amount of GNPs. As a result, the amount of the antibody’s Fc terminal adsorbed on the surface of GNPs by Au-S bonds increased, the Fab terminal of binding site to epitope is exposed to the outside. Antigen could enter the network space and have specific reactions with antibody, resulting in changes of steric hindrance. Obviously, the number of antibodies has a significant impact for sample testing parameters, e.g., concentration, sensitivity, and low limit of detection.

### 2.4. Cyclic Voltammetry and AC Impedance Graph before and after the Immune Response

**Figure 4:**
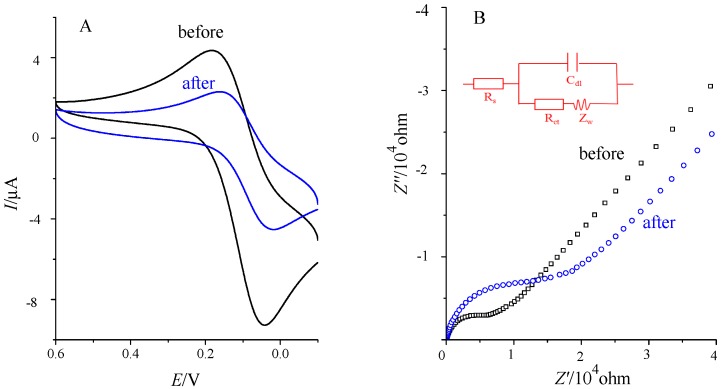
Characterization the immune response of biosensor by Cyclic Voltammetry (**A**) and AC impedance (**B**). The inset is the equivalent circuit applied to fit the impedance spectra.

Figure 4 showed the electrochemical characterization map of prepared THC immunosensor and THC before and after immune response. Figure 4A is the characterization of cyclic voltammogram (scan range of 0.6~−0.1 V and scan rate of 50 mV/s) and Figure 4B refers to the characterization of AC impedance method (10^−2^~10^6^ Hz), both of which adopted 1 mM K_3_Fe(CN)_6_ (containing 0.20 M KNO_3_) solution as the base solution. The redox peak current of cyclic voltammogram of the sensor after binding to THC decreased, and the impedance value was approximately twice what it was before the immune response. This was due to the increased steric effect of antigen-antibody immune complexes on electron transfer. The above results indicated that this immunosensor could be used in the detection of THC.

### 2.5. Optimization of Detection of Potential and Incubation Time

**Figure 5:**
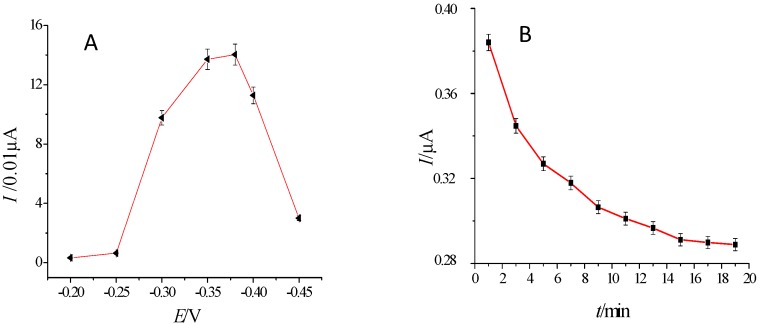
Optimization of detect potential (**A**) and incubation time (**B**).

The Amperometric I-t Curve measurement of the prepared sensor was conducted under different potentials (the base solution was 10 mL 0.01 mol/L pH 7.4 PBS + 5 µL 0.5 mol/L H_2_O_2_) as shown in Figure 5A. The difference in the steady-state current before and after incubation was used to measure the impact of different potentials on the electrochemical response of the sensor. The change value of current at −0.38 V was the maximum, and therefore −0.38V was chosen as the constant potential for the measurement.

The incubation time for the immunosensor binding to THC was optimized as shown in Figure 5B. Within 10 min of incubation, the response current drastically decreased with the increase of incubation time of the antigen and antibody. Ten min later, the decrease of the response current slowed with the increased incubation time, and the response current was basically steady 15 min later. Hence, 15 min was used as the optimum incubation time.

### 2.6. THC Quantitative Detection

**Figure 6:**
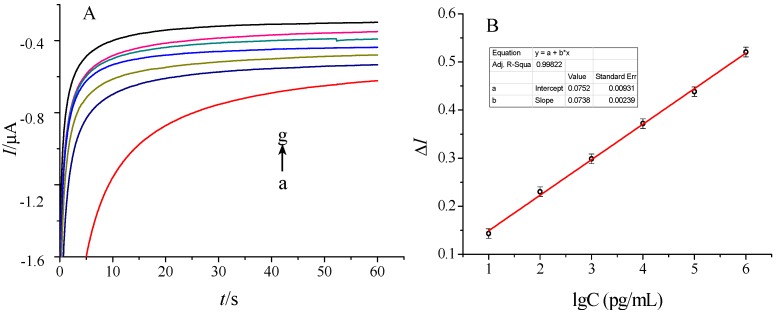
(**A**) Response curves of current for the determination of THC: a. 0.01 M pH 7.4 PBS buffer solution blank control; b~g are the multiple PBS buffer solution proportions diluted THC solution, the mass concentration is 0.01~10^3^ ng/mL; (**B**) Linear response curve for the determination of THC.

The prepared THC electrochemical immunosensor was applied to determine the THC in 0.01 M pH 7.4 PBS. The steady-state current value (at 50 s) was selected as the standard for comparison and a figure was drawn with the current variation △I between before and after immunization. Figure 6A showed the correlation between the response current of the immunosensor and THC protein concentration. Figure 6B showed a linear relationship between the change rate of the response current (µA) of the immunosensor and THC concentration within the range of 0.01~10^3^ ng/mL. The linear equation was △I = 0.07388lgC + 0.07522 (R^2^ = 0.9986), and the limit of detection (LOD) of the immunosensor was determined as 3.3 pg/mL(S/N = 3). These results indicate that the developed immunosensor can be a promising means to determine THC. Compared with other detection methods as shown in Table 1, the electrochemical immunosensor displayed a higher sensitivity and a wider linear range. There may be two reasons for this. Firstly, based on nanoscale structure and specific properties of nanomaterials, such as electricity, optics, large surface area to volume ratio, uptake by biological systems, gold nanoparticles can greatly increase the biomolecular immobilization amount (Step 2) [29,30]. Secondly, the antigen-antibody combination changed its carrier redox potential of gold nanoparticles. Then, the HRP adsorbed on the gold nanoparticles enhances the current response (Step 5). The electrical signals will be passed to CHI 660E electrochemical workstation through Thi/Chit polymer membrane (Step 4) and GNPs membrane (Step 2), and be further amplified [15].

### 2.7. Specificity, Reproducibility, and Stability of the Immunosensor

**Figure 7:**
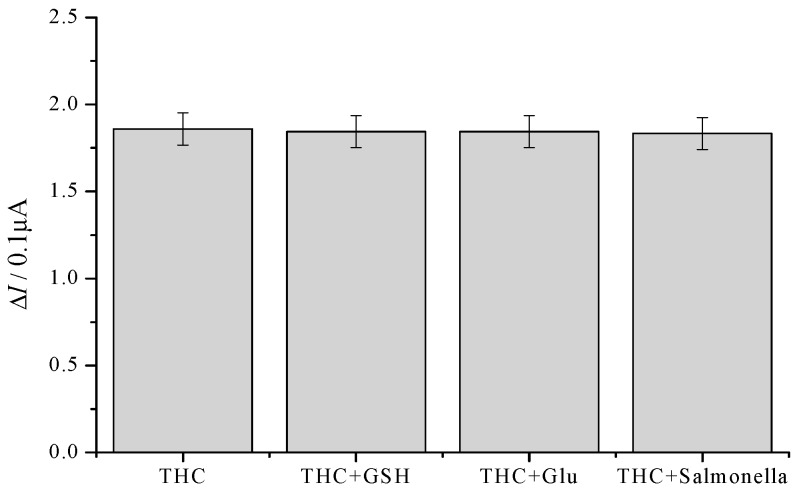
Specificity of the immunosensor to THC, THC + GSH, THC + Glu, THC + *Salmonella*, respectively. The concentration of THC, GSH, Glu, and *Salmonella* is 1 ng/mL, 1 ng/mL, 1 ng/mL, 1 × 10^3^ cfu/mL. Error bars represent percent of data 5%.

The anti-interference capability of the prepared THC electrochemical immunosensor was investigated by detecting 1 ng/mL THC in PBS with the addition of various interference species into the buffer solution, such as 1 ng/mL glutathione (GSH), 1 ng/mL glutamic (Glu) acid and 1 × 10^3^ cfu/mL *Salmonella*. Figure 7 exhibit response current of the proposed immunosensor incubated with 1 ng/mL THC, the mixture of THC and GSH, Glu, or *Salmonella* under the same experimental conditions. As can be seen from Figure 7, no significant decreasing was obtained after interfering substances were added into analyte, which indicated that the developed electrochemical immunosensor could be used to identify THC with high specificity. THC (1 ng/mL) in PBS was measured continuously by the immunosensor 12 times, with each incubating time being 15 min, and the results showed that the RSDs were 4.3%. In addition, the immunosensor was stored over 0.01 mol/L pH 7.4 PBS at 4 °C, and THC solution was detected once every three days. From day 1 to day 10, the sensor’s response current was basically constant; on day 13, the response current was 84.4% of the initial response current; on day 15, the response current was only 47.8% of the initial response current, suggesting that the immunosensor had good stability.

With five prepared THC immunosensors in different batches, 1 ng/mL THC was measured under the same conditions. The results showed the RSD of response current was 6.83%, which indicated that the immunosensor had good reproducibility.

### 2.8. Real Sample Analysis

In order to test the precision and accuracy of this proposed immunosensor, it was used to detect the recoveries of different concentrations of THC in rat serum samples by standard addition methods (Table 2) [39]. The RSD was in the range from 1.89% to 4.7% and the recovery was in the range from 98.2% to 101.5%. Thus, the designed immunosensor could be effectively applied to the quantitative detection of THC in rat serum.

## 3. Materials and Methods

### 3.1. Materials and Reagents

Chloroauric Acid was from Shenyang Jinke Reagent Factory (Shengyang, China); Sodium Citrate was from Tianjin Yingdaxi Chemical Reagent Factory; Chitosan (Chit, a degree of deacetylation ≥ 90%) was from Jinan Handebei Marine Bioengineering Co. Ltd. (Jinan, China); Bovine Serum Albumin (BSA), Tween-20 American Sigma-Aldrich Company, (St. Louis, MO, USA); 0.1 g/100 mL Chit (solution: 0.1 g Chit dissolves in 100 mL acetum with a volume fraction of 1%. Thionine Acetate (Thi), horse radish peroxidase (HRP A-1000 units/mg) were all purchased from Sigma-Aldrich; THC and anti-THC Balb/C mouse monoclonal antibody (1 mg/1 mL) were from Shanghai Yansheng Biotechnology Co. Ltd. (China). All the other reagents used were of analytical grade, and the water was ultrapure.

### 3.2. Apparatus and Facilities

KQ 3200B ultrasonic cleaner from Kunshan Ultrasonic Instruments Co., Ltd. (Kunshan, China) was used for the pretreatment of the glassy carbon electrode. CHI 660E electrochemical workstation was from Shanghai Chenhua Instrument Co., Ltd. (Shanghai, China). Three-electrode system was adopted with Ag/AgCl as the reference electrode, platinum-wire electrode as the counter electrode, and glassy carbon electrode (ϕ = 3 mm) as the working electrode. UV-2501 UV-Vis spectrophotometer was from Shimadzu (Kyoto, Japan). A Tecnai G2F20 Transmission electron microscope (TEM) from Philips was used for the characterization of gold nanoparticles under 200 KV of acceleration voltage.

### 3.3. The Preparation and Characterization of Gold Nanoparticles (GNPs)

The preparation of GNPs was conducted with reference to the previous method [40]. 100 mL of 0.01 g/100 mL chloroauric acid solution (about pH 7.0) was mixed with 4 mL of 1 g/100 mL trisodium citrate solution as the reducing agent. The resulting solution was heated for 10 min, and diluted to the original volume with the ultrapure water after being cooled to room temperature. The GNP soliquid was then obtained and stored at 4 °C for use. It was characterized by UV-Vis spectrophotometer and TEM.

### 3.4. The Preparation of the Copolymer of Thi/Chit

The preparation of the copolymer of Thi/Chit has been carried out by taking reference of the reported method in reference documentation [41] and making some improvements. Add 2.5 mL of 2% (*w/v*) Chit solution (2 g of Chit dissolve in 100 mL of 2% v/v acetic acid solution and stir for 3 h) into 320 μL of 10% glutaraldehyde (*v/v)* solution. Mixing the two solutions and add 200 μL of 0.01 mol/L Thi solution. At last, add 2% (*v/v*) acetic acid solution till the total volume is 6 mL. After mixing, the solution can be dripped or applied to the electrode, and this copolymer solution needs to be prepared for immediate use.

### 3.5. The Preparatio of GNPs/HRP

Referring to the method of Kang [42], adjust the pH of the prepared GNPs soliquid to 7.0 with 0.1 mol/L K_2_CO_3_ , then take 1 mL of GNP soliquid and 1 mL of 2.0 g/L HRP solution (0.01 mol/L pH 7.0 PBS dissolution). After being stirred for 2 h, GNPs/HRP solution was obtained.

### 3.6. The Pretreatment of Glassy Carbon Electrode (GCE)

We polished the GCE on the chamois leather with thick liquid of α-Al_2_O_3_ in particle sizes of 1.0 μm, 0.3 μm, and 0.05 μm respectively and washed the GCE in ultrasonic bath for 30 s after each polishing. The process was repeated three times and then the GCE was washed with 1:1 HNO_3_, absolute ethyl alcohol and hyperpure water, successively. In 1 mol/L of H_2_SO_4_ solution, cyclic voltammetry method was used to activate the electrode with scan range of 1.0~−1.0 V and scan rate of 100 mV/s until stable cyclic voltammetry curves appear. Record the cyclic voltammetry curves in 1 mM K_3_Fe(CN)_6_ solution (including 0.20 mol/L KNO_3_) to characterize the pretreatment effect of GCE, with scan range of 0.6~−0.1 V and scan rate of 50 mV/s. Under laboratory conditions, the peak-potential-differences of cyclic voltammetry curve after pretreatment should be below 80 mV, and should be as close to 64 mV as possible. Only under this condition can the electrode be used. At last, it is set aside to dry in the nitrogen environment for later use.

### 3.7. The Preparation of the Immunosensor

**Figure 8:**
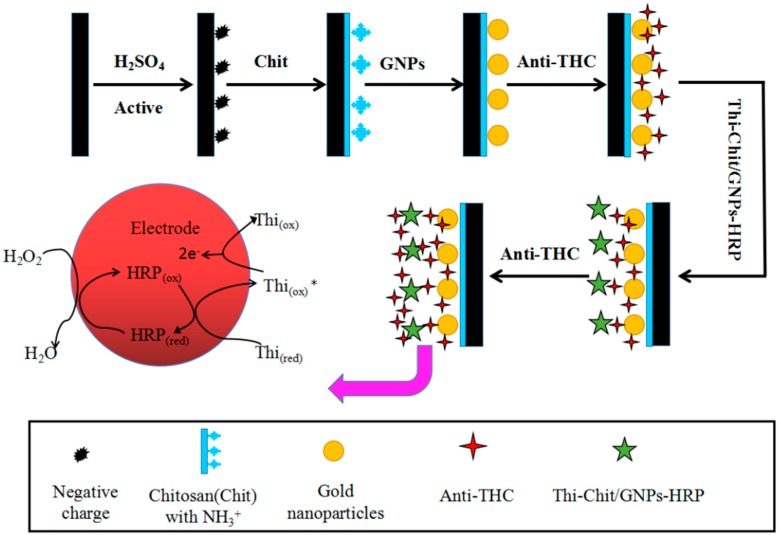
Schematic illustration of THC immunosensor fabrication procedures.

Referring to the electrochemical immunosensor signal amplification system of the previously reported method [43,44], the preparation steps are as shown in Figure 8 below. After pretreating GCE, take 5 μL of 0.5% Chit solution (dissolved in 1% acetic acid solution) and drip it on the surface of the electrode. Place it to dry in the oven at 45 °C for 3 h. When it cools to room temperature, sink it in 1 mol/L NaOH solution for 5 min, then clean it with hyperpure water and sink it in hyperpure water for 30 min (step 1); take it out for natural drying and place it in the GNP soliquid for 24 h (Step 2); then, place the electrode in 0.5 mg/mL anti-THC monoclonal antibody solution at 4 °C for self-assembly for 24 h, thus obtaining the sensor with monolayered GNPs modification (Step 3); take 5 μL of the copolymer solution of Thi/Chit and drip it onto the center of surface of the above electrode. After it is naturally dried, wash the polymer film repeatedly with hyperpure water until the washed water does not have a light absorption value under 600 nm. Then, place the electrode in the GNPs/HRP solution at 4 °C for self-assembly for 24 h (Step 4); after washing with hyperpure water, place the electrode in anti-THC monoclonal antibody solution again at 4 °C for self-assembly for 24 h (Step 5); at last, place the modified electrode in BSA solution (1 g/100 mL) at 37 °C for incubation for 1 h to close unspecific points and wash the unconjugated BSA with PBST solution that contains 0.05% (*v/v*) Tween-20 (Step 6); the Electrochemistry Nano Immunosensor can be obtained after being dried naturally, which is set aside in the PBS buffer environment at 4 °C for later use.

In the presence of H_2_O_2_, the HRP and Thi immobilized on the GCE could result in a series of redox reactions as follows:

HRP + H_2_O_2_→Compound + H_2_O; (1)

Compound + Thionine (red)→Compound +Thionine (ox)*;(2)

Compound + Thionine (ox)* + 2H^+^→HRP + Thionine (ox) + H_2_O; (3)

Thionine (ox) + 2e^−^ + 2H^+^→Thionine (red). (4)

The immune complex formed through antibody-antigen binding on the electrode surface could hinder the transfer of electron due to steric hindrance effect, and the changes of steric hindrance can be determined based on the current change. Thus, we can determine the sample concentration by measuring the response current change resulting from before and after the immune reaction.

### 3.8. The Determination Method of the Immunosensor

The three-electrode system was used with GCE was as the working electrode, Ag/AgCl electrode as the reference electrode, Pt electrode as the control electrode, and the 0.01 mol/L pH 7.0 PBS buffer solution + 5 µL 0.5 mol/L H_2_O_2_ as the base solution. The scanning was conducted at −0.38 V constant potential to get the current-time curve, and the change of the steady currents (I1 and I2) before and after immune response was adopted for quantitative detection of the THC.

## 4. Conclusions

In this study, a novel double-layer gold nanoparticle-modified electrochemical immunosensor was developed for detection of THC. The electrode was fabricated with an electrochemical biosensing signal amplification system in absorption of gold nanoparticle-thionine-chitosan for HRP, and chitosan cross-linking electron mediator thionine with a good biocompatibility and film formation for absorption of gold nanoparticles. The immunosensor was validated with wide detection concentration range, high sensitivity, selectivity and reproducibility, fast response, and good stability, which provided an efficient means for determining THC.

## Figures and Tables

**Table 1:** Comparison of the performances of some different THC detection methods.

Detection Method	Linear Range	LOD	Detection Time	Reference
One-Step^TM^-ELISA	0.1–6.5 ng/mg	0.1 ng/mg	≈2 h	[31]
LUCIO^®^-Direct-ELISA	≥10 ng/mL	2.5 ng/mL	≈2 h	[32]
LC-ESI-MS^3^	0.13–15.75 pg/mg	0.05 pg/mg		[33]
GC-MS	0.16–2.3 ng/mg	0.1 ng/mg	>1 h	[34]
HPLC-UV	10–10^4^ ng/mL	10 ng/mL	>20 min	[35]
Microwave-Accelerated Derivatization and GC-MS	5–100 ng/mL	5 ng/mL	≈30 min	[36]
Field Asymmetric Ion Mobility Spectrometry Microchip Sensor (FAIMS)	6.5–40 ng/mg	6.5 ng/mg		[37]
On-Line Stacking Capillary Electrophoresis	0.04–6 µg/ml	10 ng/mL		[38]
Electrochemical Nano Immunosensor	0.01–10^3^ ng/mL	3.3 pg/ml	≈10 min	This work

**Table 2:** Determination of THC in rat serum sample.

Initial THC Concentration in Sample (ng/mL)	Added THC Concentration (ng/mL)	Measured Concentration after Addition (ng/mL)	RSD (%, *n* = 5)	Recovery (%, *n* = 5)
1	1	2.03, 2.12, 2.2, 2.07, 1.94	4.7	100.6
	5	6.02, 5.97, 6.16, 6.24, 6.19	1.89	101.5
	10	10.98, 11.46, 10.92, 12.11, 11.07	4.38	98.2
